# Co-culture of JEG-3, BeWo and syncBeWo cell lines with adrenal H295R cell line: an alternative model for examining endocrine and metabolic properties of the fetoplacental unit

**DOI:** 10.1007/s10616-017-0142-z

**Published:** 2017-09-30

**Authors:** Eliza Drwal, Agnieszka Rak, Ewa Gregoraszczuk

**Affiliations:** Department of Physiology and Toxicology of Reproduction, Institute of Zoology and Biomedical Research, Jagiellonian University in Krakow, Gronostajowa 9, 30-387 Kraków, Poland

**Keywords:** Placenta cell lines, H295R cells, Fetoplacental steroidogenesis, Fetoplacental metabolism, Receptors expression

## Abstract

Monocultures of different placental cells are used for many physiological and toxicological studies; however, they are not a true reflection of the interaction between placenta and fetus. To develop the most appropriate model to study endocrine and metabolic properties of fetoplacental unit we used three co-culture models of placental cells nonfusogenic JEG-3, unsyncytialised BeWo (BeWo) and syncytialised BeWo (syncBeWo) cultured with adrenal (H295R) cells. As an end point of endocrine properties we investigated steroids receptors expression and steroid secretion, while as metabolic properties AhR, CYP1A1and COMT expression. Progesterone (P4), estradiol (E2) and human chorionic gonadotropin (hCG) secretion (ELISA) and 3βHSD, CYP19, estrogen (ERα/β), progesterone (PR) and aryl hydrocarbon (AhR) receptors, CYP1A1 and COMT protein expression (Western blot) were evaluated. Comparing three co-culture models we observed: (1) there were no differences between JEG-3 and BeWo in the PR expression, however it was higher in BeWo compared to syncBeWo; (2) there were no differences in ERα protein expression in all models, while profile of ERβ expression was the highest in syncBeWo; (3) high P4 secretion in JEG-3 and BeWo while low in syncBeWo; (4) high E2 levels in JEG-3 and syncBeWo, while low E2 secretion in BeWo; (5) the highest hCG secretion in the JEG-3 and syncBeWo than in BeWo (6) the highest AhR, CYP1A1 and COMT expression in syncBeWo. Based on the results showing higher hCG secretion in the JEG-3 than in BeWo, representing villous and extravillous phenotype we suggest that JEG-3 model could be used to study fetoplacental steroidogenesis at the 1st, while BeWo model at the 3rd. Results showing comparable profiles of AhR, CYP1A1 and COMT expression in JEG-3 and BeWo models and the significantly higher expression in synBeWo points to synBeWo as a good model for study the metabolic properties.

**Electronic supplementary material:**

The online version of this article (doi:10.1007/s10616-017-0142-z) contains supplementary material, which is available to authorized users.

## Introduction

Cell-lines derived from human placenta and chorion, such as human choriocarcinoma cells BeWo or JEG-3 are an alternative for study the placenta function (Sullivan 2002). There are two main differentiation pathways in human placentas, villous (VCT) and extravillous (EVT) cytotrophoblast (Lee et al. 2016). BeWo represent placenta villous (Orendi et al. 2010), while JEG-3 extravillous cells (Lee et al. 2016). Microarray analysis has indicated that approximately 2700 genes are differentially expressed between BeWo and JEG-3 cells, suggesting that they are suited only for specific experimental paradigms. These differences in gene expression patterns suggest that JEG3 and BeWo cell lines will vary in their capacity to respond to an identical experimental treatment (Burleigh et al. 2007). JEG-3 lines are widely used to study the molecular mechanisms underlying the proliferation and invasive potential of cytotrophoblast, while BeWo cells are commonly used to study syncytialisation, adhesion and endocrine function (Hannan et al. 2010). Importantly, BeWo cell lines have fusogenic properties and, in culture with forskolin (a cyclic AMP inducer), are able to form syncytiotrophoblasts (Wice et al. 1990), thus making them a good third trimester placental model (Zachariades et al. 2011). Moreover, few papers indicate that significant differences in gene expression promotes fusion such as: β subunit of human chorionic gonadotropin, placental alkaline phosphatase, LGALS13, syncytin-1 or syncytin-2 in BeWo cells treatment with forskolin compared to non-treated BeWo cells (Orendi et al. 2010; Frendo et al. 2003).

In the complex scenario of the feto–maternal interface, endocrine, paracrine, and autocrine factors must be taken into consideration. Hormones, such as E2, P4 and hCG are the major players of the endocrine regulation that take place at the feto–maternal interface. hCG, which is the hormone produced by the trophoblast in the very early stages of pregnancy, plays a key role in making sure that the endometrium is ready to receive the embryo implantation (Tsampalas et al. 2010). The placenta has poor capacity of producing de novo estrogen (Escobar et al. 2011), therefore uses androgens such as dehydroepiandrosterone (DHEA), derived from the fetal and maternal adrenal glands to produce estrogen (Gude et al. 2004). Experimental models using independent cell lines alone do not reflect the interaction between placenta and fetus and provide only partial information concerning hormone secretion; moreover, when cultured alone, cell lines require supplementation with precursors for steroid secretion. The H295R human adrenocortical carcinoma cell line possesses all the enzymatic capacities of the undifferentiated fetal adrenal gland, and produces androgens, therefore providing fetal precursors for estrogen (Thibeault et al. 2014). The expression of PR in BeWo, syncBeWo and JEG-3 cell lines was described by Zachariades et al. (2011). Furthermore, two isoforms of ERs in BeWo cells (Jiang et al. 1997) and JEG-3 cells have also been demonstrated (Mehta et al. 2002).

Additionally, the placenta-expressed enzymes of phase I (i.e. CYP1A1) and phase II (i.e. catechol-O-methyl transferase, COMT) of metabolism are responsible for the detoxification of endogenous and exogenous hormones (Isoherranen and Thummel 2013; Hakkola et al. 1996a). CYP1A1 expression and activity have been detected in the first and third trimesters at both mRNA and protein levels (Hakkola et al. 1996b; Stejskalova and Pavek 2011; Czekaj et al. 2005). There are data showing that both placental (Hakkola et al. 1996b; Stejskalova et al. 2013) and adrenal (Bláha et al. 2006) CYP1A1 are regulated by AhR. COMT activity in the full-term placenta has been described by Barnea et al. (1988). To our knowledge there are no data showing COMT protein expression in placenta cell lines.

Coculture models including cells and/or tissues representative of both the fetus and the maternal counterpart have been developed to study a wider spectrum of interactions at the feto–maternal interface (Wang et al. 2012; Dunk et al. 2003; Helige et al. 2008; Moser et al. 2010). Such approaches mainly focus on the invasion of human placenta inside the maternal decidua. These coculture models are a powerful tool as they include all the main cell types involved at the feto–maternal interface. They nevertheless require synchronization of primary cultures from tissues which are sometimes difficult to obtain. In order to overcome these limitations, Mannelli et al. (2015) recently applied an in vitro system that could be helpful to study the molecular interactions at the feto–maternal interface even in laboratories that do not have availability of fresh tissues. The co-culture of H295R and BeWo cells as a unique in vitro model to reproduce the steroidogenic cooperation between fetus and placenta during pregnancy have been characterized and proposed for use to study the endocrine-disrupting effects of environmental chemicals by Thibeault et al. (2014). As an end point authors analyzed progesterone, DHEA, androstenedione, estradiol, estriol, and estrone, CYP 19 activity and hCG production by H295R and BeWo cells in monoculture or in co-culture over a 24 h period.

In the presented data we decided despite of BeWo cell line, to use JEG-3 cells with non-fusogenic properties and syncBeWo representing syncytiotrophoblast and additionally as an end point CYP19 and 3β-HSD protein expression, steroid receptors (ERα/β, PR) protein expression, while for metabolic properties we selected AhR, CYP1A1 and COMT protein expression.

## Materials and methods

### Reagents

DMEM/F12 medium, phosphate buffered saline (PBS), trypsin were purchased from Gibco Life Technologies (Paisley, United Kingdom). Fetal bovine serum (FBS, heat inactivated and stripped) was purchased from Biowest (Nuaillé, France). NuSerum and ITS + Premix were obtained from BD Biosciences (Mississauga, Ontario, Canada). Insulin, glycerol, ethylenediaminetetra-acetic acid (EDTA), dithiothreitol (DTT), bromophenol blue, Na-deoxycholate, Nonidet NP-40, protease inhibitors (EDTA-free), Tween 20, ammonium persulfate (APS), N,N,N′N′-tetramethylethylene-diamine (TEMED) were obtained from Sigma-Aldrich (St. Louis, MO, USA). Tris base, NaCl, sodium dodecyl sulfate (SDS), bovine serum albumin (BSA) were purchased from Bioshop Canada Inc. (Burlington, ON, Canada). Methanol and HCl were obtained from Avantor Performance Materials (Gliwice, Poland). WesternBright™Sirius Western blotting detection kit was obtained from Advansta (Menlo Park, CA, USA). A Bradford protein assay kit was obtained from Bio-Rad Laboratories (Hercules, CA, USA). Polyvinylidene difluoride (PVDF) membrane was purchased from Merck Millipore (Darmstadt, Germany). Marker of electrophoresis Thermo Scientific Page Ruler Prestained Protein Ladder was purchased from Thermo Fisher Scientific (Waltham, MA, USA). Antibodies were listed in Table 1.

**Table 1:** Primary and secondary antibodies

Name	Cat no.	Dillution	Company
*Primary antibodies*			
3βHSD goat polyclonal	#30820	1:200	Santa Cruz Biotechnology (Danvers, MA, USA)
CYP19 goat polyclonal	#14244		
CYP1A1 goat polyclonal	#9828		
COMT rabbit polyclonal	#25844		
AhR goat polyclonal	#8088		
ERα rabbit polyclonal	#544		
ERβ rabbit polyclonal	#8974		
PR A/B rabbit polyclonal	#3176S		
β-actin rabbit monoclonal	#A5316	1:2000	Sigma Aldrich (St. Louis, MO, USA)
*Secondary antibodies*			
Anti-goat horseradish peroxidase-conjugated	#sc2020	1:1000	Santa Cruz Biotechnology (Danvers, MA, USA)
Anti-rabbit horseradish peroxidase-conjugated	#7074	1:1000	Cell Signaling Technologies (Leiden, Netherlands)

### Cell culture

BeWo (cat. CCL-98, ATCC, Manassas, VA, USA) passages 18–22, JEG-3 (cat. HTB-36, ATTC) passages 1–6, and H295R (cat. 300483, CLS, Eppelheim, Germany) passages 17–21 were used in the experiments. All cell lines were cultured in DMEM/F12 without phenol red, supplemented for BeWo cells with: 0.01 mg/ml insulin and 10% heat-inactivated FBS, with 10% heat-inactivated FBS for JEG-3 cells and with 2.5% NuSerum and 1% ITS + Premix for H295R cells. BeWo cells have been cultured as non-differentiated (BeWo) and differentiated (sync. BeWo) cells. Differentiation was achieved using 50 µM forskolin. Dose of forskolin was selected based on preliminary experiment with concentrations of forskolin from 10 to 100 µM and data published by Zachariades et al. (2011). To select appropriate dose of forskolin effect on proliferation/cytotoxicity and hormone secretion was examined using Alamar blue assay and ELISA (data not shown). Firstly BeWo were cultured in flasks treated with forskolin for 72 h and then seeded on a plate like syncBeWo. Differentiation markers like syncytin-1 mRNA expression and hCG secretion were chosen based on data previously published by Huang et al. (2009) and analyzed before and after trypsinization. Moreover, viability was measured using Alamar Blue test.

### Experimental procedure

H295R, BeWo, syncBeWo or JEG-3 (12.5 × 10^4^ cells/200μl/well in 96-well) were cultured alone for 24, 48, and  72 h. At the end of culture, media were frozen at −20 °C for further hormone levels analysis while cells were lysed with lysis buffer, sonicated and centrifuged at 15,000×*g* for 15 min at 4 °C and stored at −20 °C for Western blot analysis.

**Fig. 1:**
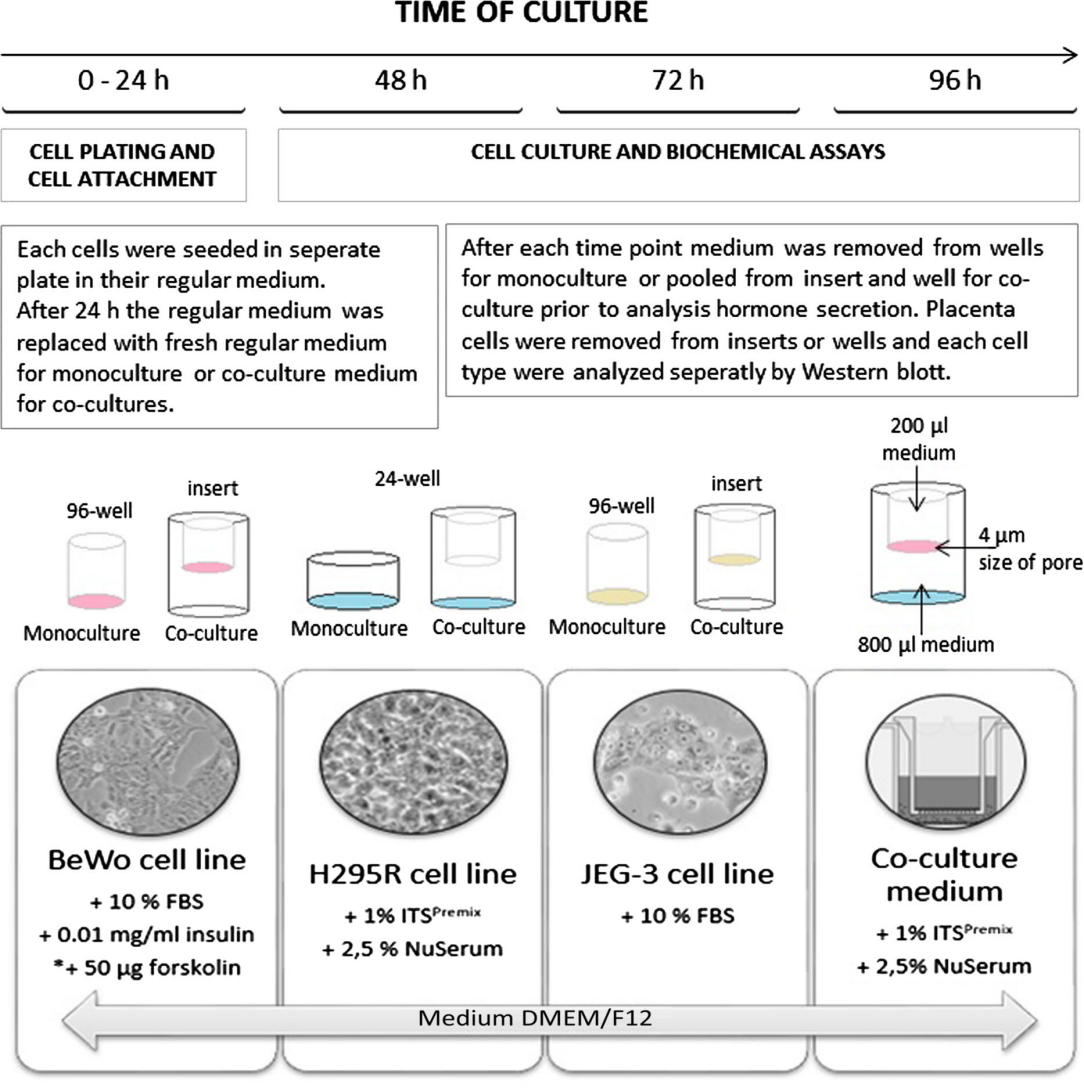
Experimental procedure

In co-culture BeWo, syncBeWo or JEG-3 cells (1.25 × 10^4^ cells/200μl/well) were cultured on 0.4 µm pores/insert with H295R cells (2.5 × 10^4^ cells/800 μl/well) in 24-well for 72 h. 24 h after seeding inserts with BeWo or JEG-3 cells were placed into the wells with H295R cells and media were replaced with fresh H295R cell medium. Hormone levels in medium were evaluated after 24, 48, and 72 h. For co-culture, medium was pooled from inserts and wells. At the end of the incubation period, the cells from inserts (BeWo or JEG-3) were washed with ice-cold PBS, lysed with lysis buffer, sonicated and centrifuged at 15,000×*g* for 15 min at 4 °C and stored at −20 °C for Western blot analysis. Protein expression was determined in placenta cells (from wells for monoculture or inserts for co-culture) (Fig. 1)

Cell density for both types of culture was determined experimentally in preliminary studies with 3–15 × 10^4^ for placental cells and 20–100 × 10^4^ for adrenal cells considering size of the plates, time of culture, cell proliferation and hormone secretion.

### Real time PCR

After 24 and 72 h of incubation with PAHs, the cells were washed with ice-cold PBS, and the plates were frozen at −20 °C until needed for the performance of PCR analysis. Total RNA isolation and cDNA synthesis was performed using the TaqMan Gene Expression Cell-to-CT Kit (Applied Biosystems, Carlsbad, CA, USA) following the manufacturer’s protocol. The purity and quantity of the RNA and cDNA were determined using spectrophotometry at two optical densities, 260 and 280 nm (DeNovix DS-11 Spectrophotometer, Wilmington, DE, USA). Amplifications were performed using the StepOnePlus system (Applied Biosystems, Carlsbad, CA, USA) and the TaqMan syncytin-1 in combination with the TaqMan Gene Expression Master Mix (Applied Biosystems), following the manufacturer’s instructions. PCR was performed using a final volume of 20 μl, including 50 ng/reaction cDNA. The PCR conditions were performed as follows: pre-incubation for 2 min at 50 °C and 10 min at 95 °C, amplification for 40 cycles (15 s at 95 °C and 1 min at 60 °C). The relative expression of genes was normalized against the endogenous reference gene GAPDH (Human GAPD Endogenous Control, Number 4333764F); ΔC_q_) and converted to relative expression (RQ) using the 2^−ΔΔCq^ method. The results are expressed in the figures as relative values (RQ).

### Alamar blue assay

The alamarBlue assay (Invitrogen) is designed to measure the proliferation of different cell types and is based on quantitation of the cell’s metabolic activity. Cellular metabolism induces a chemical reduction of the alamarBlue medium, i.e., this assay is based on the quantitative metabolic conversion of the blue, nonfluorescent resazurin to pink, fluorescent resorufin by living cells. AlamarBlue has minimal cell toxicity and is therefore appropriate for continuous monitoring of proliferation in the same cell culture at various time points. AlamarBlue stock solution was aseptically added to the wells in amounts equal to 10% of the incubation volume. The resazurin reduction in the cultures after 24 and 72 h of treatment with PAHs was determined after 3 h incubation with alamarBlue by measuring the absorbance at 570 nm and 600 nm wavelengths using a FLUORO microplate reader (BioTek Instruments, Winooski, VT, USA). The results are expressed in the figures as relative fluorescence *units* (RFU).

### ELISA

P4, E2 and hCG levels were determined using commercially available ELISA kits (cat. EIA-1561, EIA-2693 and EIA-1911, respectively, DRG Instruments GmbH, Marburg, Germany). For P4 the sensitivity of assay was 0.045 ng/ml. The inter- and intra-experiment coefficients of variation were 4.34 and 6.99%, respectively. Cross reactivity with P4: 100%, 17αOH progesterone: 0.30%, pregnenolone: 0.35%, estriol: <0.10% and 17αestradiol: <0.10%. For E2 the sensitivity of assay was 9.714 pg/ml. The inter- and intra-experiment coefficients of variation were 6.72 and 2.71%, respectively. Cross reactivity of the E2 assay were tested for E2: 100%, estriol: 0.05%, estrone: 0.2%, P4: 0% and pregnenolone 0%. For hCG the sensitivity of assay was 1 mlU/with ranges of 0–200 mlU/ml. The inter- and intra-experiment coefficients of variation were 7.3 and 4%, respectively.

### Western blot

80 µg of protein (determined by the Bradford method) from each treatment group were separated by 10% SDS-PAGE and transferred to polyvinylidene difluoride membranes. The blots were blocked for 1 h in 5% w/v BSA and 0.1% v/v Tween 20 in 0.02 M TBS buffer and incubated with primary. The blots were then incubated with primary antibodies listed in Table 1 diluted 1:200 at 4 °C overnight and then with a horseradish peroxidase-conjugated secondary antibody diluted 1:1000. After visualization, blots were stripped for 30 min using stripping buffer (glycine, HCl 1% SDS), next blocked 1 h in 5% w/v BSA and 0.1% v/v Tween 20 in 0.02 M TBS buffer and incubated with primary antibodies for anti-β-actin diluted 1:2000 at 4 °C overnight and then with a horseradish peroxidase-conjugated secondary antibody diluted 1:1000. Signals were detected by chemiluminescence using a WesternBright™Sirius, and visualized using a ChemiDoc-It Imaging System.

### Statistical analysis

All samples were analyzed in triplicate in the same assay and all assays were repeated three times (n = 3). Experimental results are presented as mean ± S.D. All statistical analyses were performed using Graph Pad Prism 5. The data were analyzed by one- or two-way ANOVA followed by Tukey’s honest significant differences multiple comparison test. Statistical significant differences between time of culture and mono versus coculture are indicated with different letters a;b;c;d;e (*P* < 0.05), the same letters indicating no significant differences.

## Results

### Syncytin-1 gene expression in BeWo and syncBeWo before and after trypsynization

Before trypsynization, forskolin increased 23 fold syncytin-1 gene expression (*P* < *0.001*) (Table 1, Suppl). After trypsynization syncytin-1 gene expression was 37 fold higher compared to BeWo not treated with forskolin (*P* < *0.001*) (Table 1 in Supplementary).

### Effect of trypsynization on syncBeWo cell’s viability

24 and 72 h after trypsynization, in both, monoculture of syncBeWo and co-culture of syncBeWo with H295R cells no effect on cell proliferation was observed (Fig. 1 Suppl).

### Hormone secretion and steroidogenic enzymes expression

**Fig. 2:**
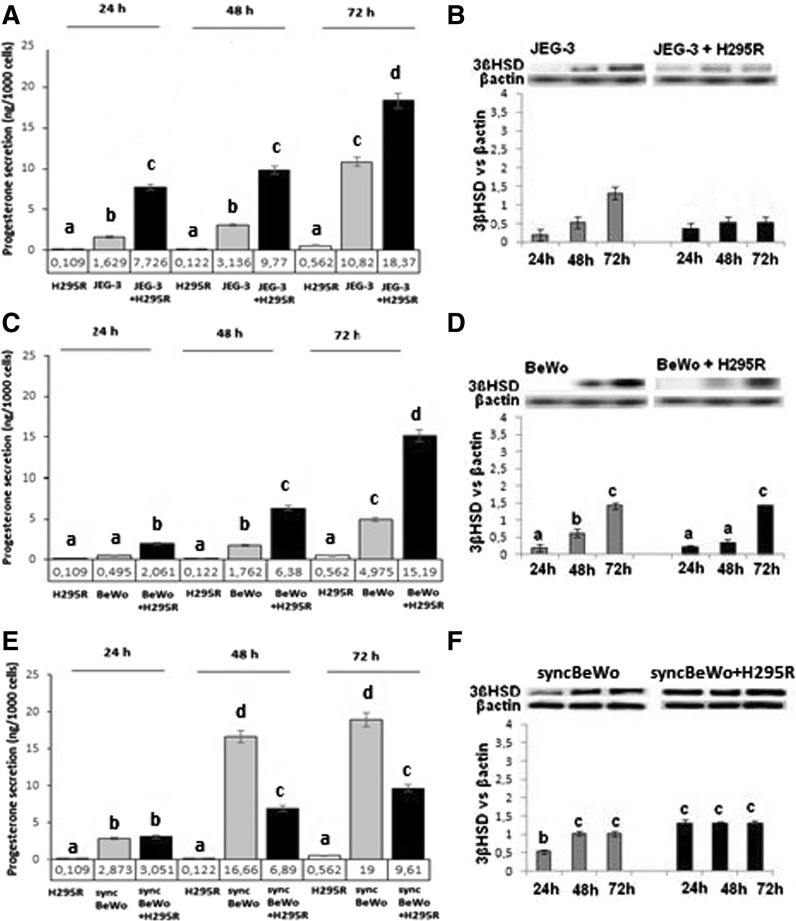
P4 secretion and 3βHSD protein expression in JEG-3 (**A**, **B**), BeWo (**C**, **D**) and syncBeWo (**E**, **F**) mono- and co-cultures with H295R. The protein levels of 3βHSD (42 kDa) were densitometrically scanned and normalised against the β-actin (42 kDa) signal. ELISA and Western blotting experiments were independently performed and repeated three times (n = 3). The data are plotted as the mean ± S.D. Statistical significant differences between time of culture and mono- versus co-culture are indicated with different letters (*P* < 0.05), the same letters indicating no significant differences

**Fig. 3:**
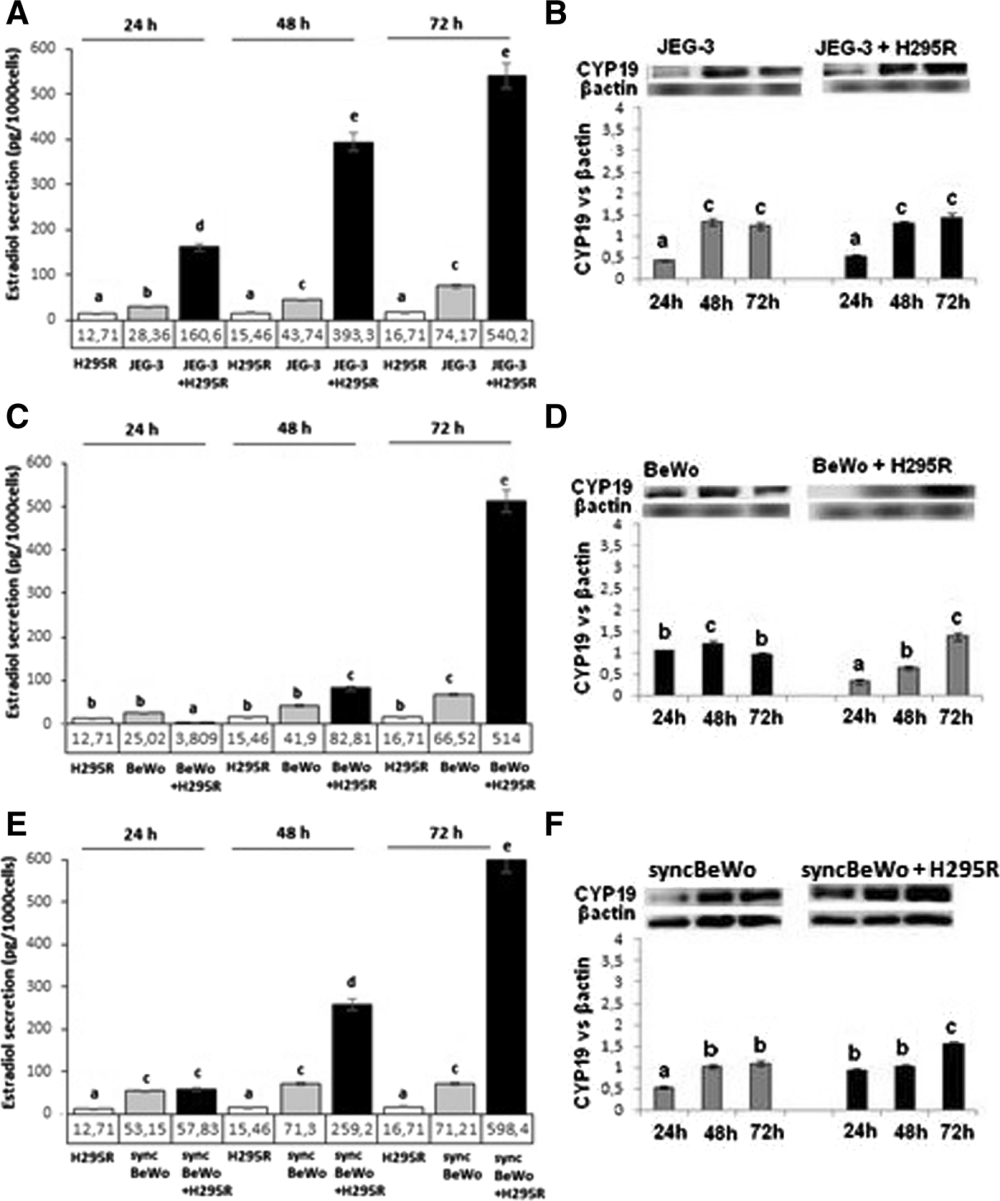
E2 secretion and CYP19 protein expression in JEG-3 (**A**, **B**), BeWo (**C**, **D**) and syncBeWo (**E, F**) mono- and co-cultures with H295R. The protein levels of CYP19 (50 kDa), were densitometrically scanned and normalised against the β-actin (42 kDa) signal. ELISA and Western blotting experiments were independently performed and repeated three times (n = 3). The data are plotted as the mean ± S.D. Statistical significant differences between time of culture and mono versus coculture are indicated with different letters (*P* < 0.05), the same letters indicating no significant differences

H295R cells secreted low amount of P4 (Fig. 2A, C, E) and E2 (Fig. 3A, C, E). In co-cultures of JEG-3 and BeWo with H295R cells' P4 secretion was higher than in the monoculture while in syncBeWo lower versus monoculture (*P* < *0.05*) (Fig. 2A, C, E). A similar pattern was observed for 3βHSD protein expression in mono- but not in co-culture with JEG-3 cells (*P* < *0.05*) (Fig. 2B) and in both mono and co-culture with BeWo (*P* < *0.05*) (Fig. 2D). In syncBeWo an increase in 3βHSD protein expression in monoculture (*P* < *0.05*) and no difference in co-culture was noted (Fig. 2F).

E2 secretion in monoculture of all JEG-3, BeWo and sync BeWo increased with time of culture. In JEG-3 with H295R cells co-culture 17, 27 and 21-fold higher levels of E2 were observed after 24, 48 and 72 h, respectively, and was paralleled with increased CYP19 protein expression (*P* < 0.05) (Fig. 3B). In the BeWo co-culture model a time dependent effect was noted compared to monoculture: both E2 secretion and CYP19 expression decreased after 24 h of culture, increased E2 secretion with decreased CYP19 expression after 48 h of culture and 23-fold higher E2 secretion corresponding with increased CYP19 expression after 72 h of culture was noted (Fig. 3C, D). In syncBeWo cells co-culture, E2 levels significantly increased and was 3.2, 10, and 25-fold higher after 24, 48 and 72 h, respectively, versus monoculture (*P* < *0.05*) (Fig. 3E). Significantly increased CYP19 protein expression in both mono- (after 48 h) and co-culture (after 72 h) was observed (*P* < *0.05*) (Fig. 3F).

**Fig. 4:**
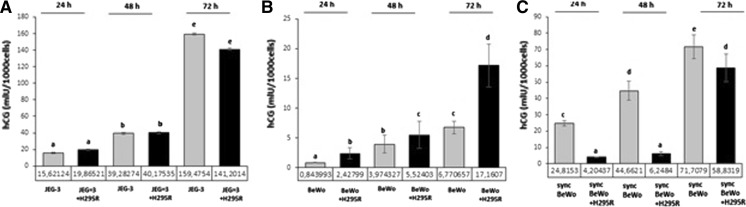
hCG secretion in JEG-3 (**A**), BeWo (**B**) and syncBeWo (**C**) in mono- and co-cultures with H295R. The data are plotted as the mean ± SD. Statistical significant differences between time of culture and mono versus coculture are indicated with different letters (*P* < 0.05), the same letters indicating no significant differences

Levels of hCG significantly increased with time of culture in monoculture of all investigated cell lines (Fig. 4). There was no change in levels with time in co-culture of JEG-3 with H295R, 3.0, 1.4 and 2.5-fold higher after 24, 48 and 72 h, respectively, of co-culture of BeWo with H295R (*P* < 0.05) (Fig. 4A, B), while in co-culture of sync BeWo with H295R 5.9, 7.1 and 1.2-fold lower levels were observed at 24, 48 and 72 h, respectively (*P* < 0.05) (Fig. 4C).

**Fig. 5:**
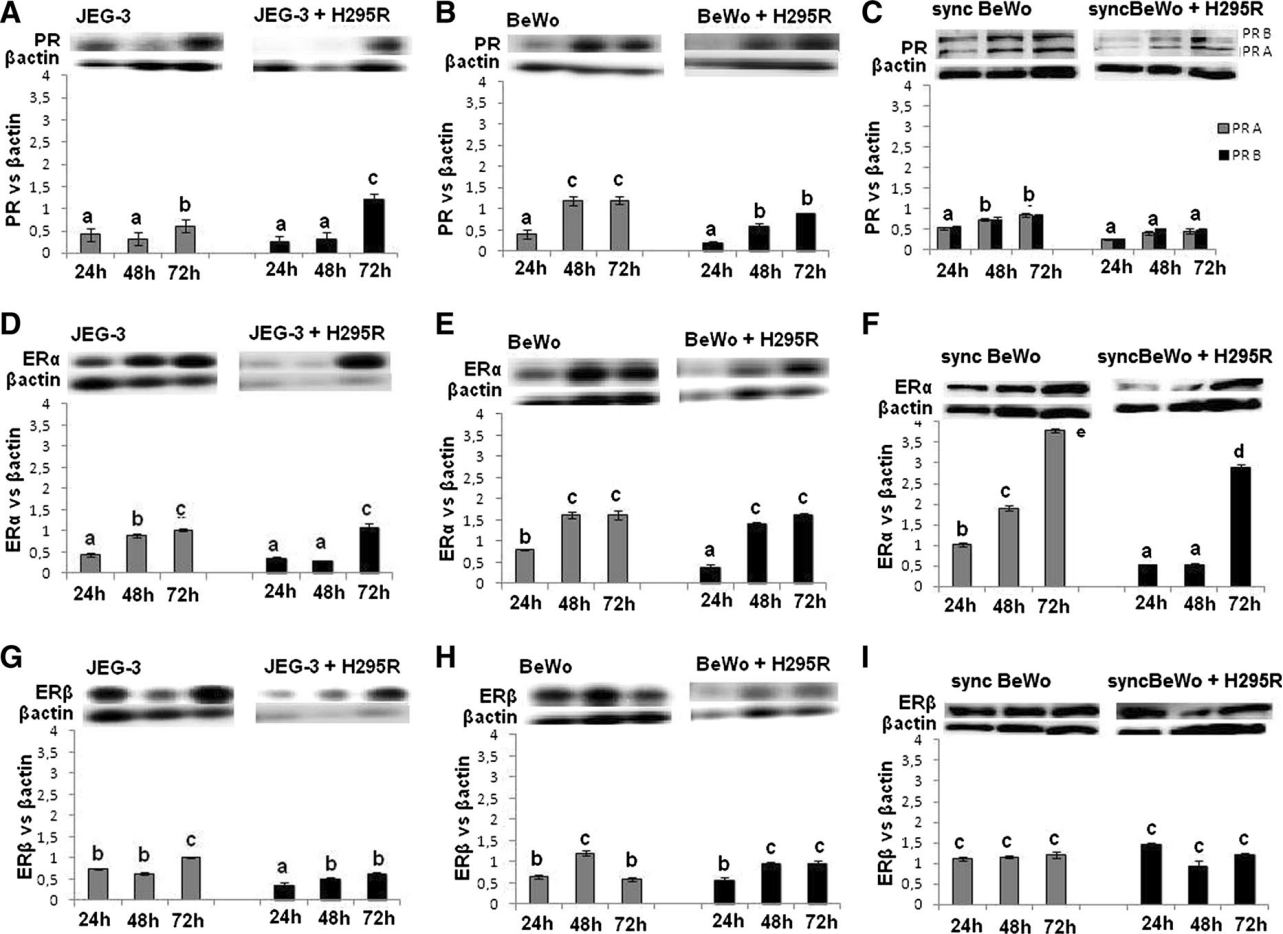
Expression of PR A/B and ERα/β in JEG-3 (**A**, **D**, **G**), BeWo (**B**, **E**, **H**) and syncBeWo (**C**, **F**, **I**) in mono- and co-cultures with H295R. The protein levels of PR (PR A 118 kDa; PR B 90 kDa), ERα (65 kDa), and ERβ (55 kDa) were densitometrically scanned and normalised against the β-actin (42 kDa) signal. Western blotting experiments were independently performed and repeated three times (n = 3). The data are plotted as the mean ± S.D. Statistical significant differences between time of culture and mono- versus coculture are indicated with different letters (*P* < 0.05), the same letters indicating no significant differences

PR protein expression in monoculture was higher in BeWo than in JEG-3 cells and higher in BeWo than in syncBeWo cells. The profile of PR protein expression, in both mono and co-culture of JEG-3 and BeWo cells, increased during time of cell culture, while no changes were observed in syncBeWo cells (*P* < *0.05*) (Fig. 5A–C). Interestingly, although antibodies against total PR were used, in fully syncytialised BeWo cells (treated with 50 μM forskolin for 72 h) two forms of  PR (A + B) were observed.

The profile of ERα protein expression increased with time of culture (*P* < *0.05*) (Fig. 5D–F) in all tested models; however, expression was significantly higher in syncBeWo cells in mono- and co-culture with H295R cells (*P* < *0.05*) (Fig. 4F). The profile of ERβ in mono- and co-culture of JEG-3 and BeWo cells was comparable and increased with time of culture, while in syncBeWo cells there was no change in levels during all times of culture. (*P* < *0.05*) (Fig. 5G–I).

**Fig. 6:**
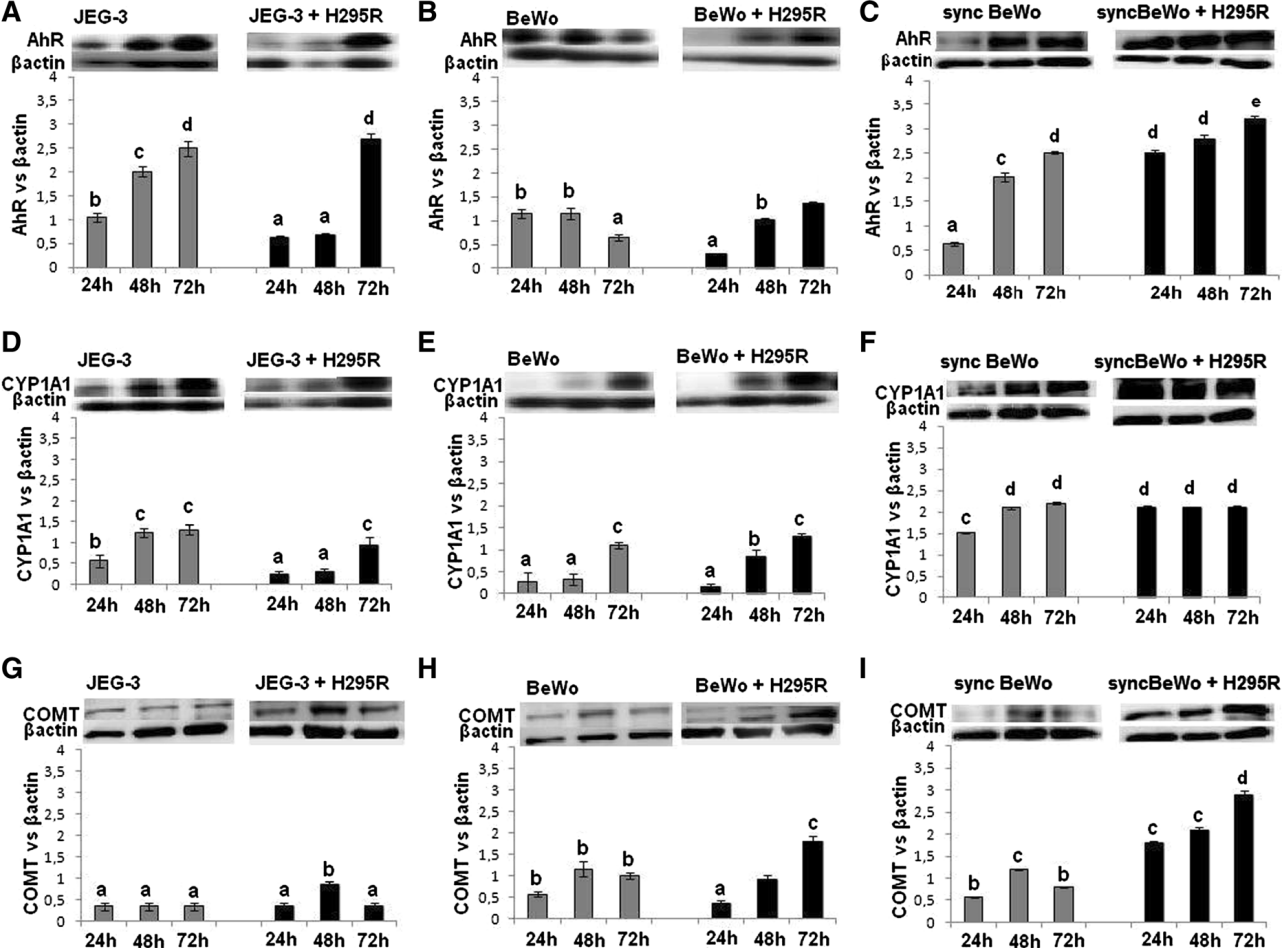
Protein expression of AhR, CYP1A1 and COMT in JEG-3 (**A**, **D**, **G**), BeWo (**B**, **E**, **H**) and syncBeWo (**C**, **F**, **I**) in mono- and co-culture with H295R. The data are plotted as the mean ± SD. The protein levels of AhR (90 kDa), CYP1A1 (56 kDa), COMT (25 kDa) were densitometrically scanned and normalised against the β-actin (42 kDa) signal. Western blotting experiments were independently performed and repeated three times (n = 3). Statistical significant differences between time of culture and mono- versus coculture are indicated with different letters (*P* < 0.05), the same letters indicating no significant differences

The expression of AhR in JEG-3 cells increased with time of culture both in mono- and co-culture with H295R cells and reached the highest level after 72 h of culture (Fig. 6A). In monoculture of BeWo cells, AhR protein expression decreased after 72 h of culture while in co-culture expression was lower at 24 h in comparison with monoculture, and at the same levels at 48 and 72 h, as in monoculture (*P* < *0.05*) (Fig. 6B). In monoculture of syncBeWo cells, AhR protein expression increased with time of culture and was significantly higher than in monoculture of BeWo cells. In co-culture of syncBeWo cells with H295R cells, high expression was noted during all times of culture and this was twofold higher than in co-culture of BeWo cells with H295R (*P* < *0.05*) (Fig. 6C).

The profile of CYP1A1 protein expression in monoculture and co-culture with H295R cells in JEG-3 cells and BeWo cells was comparable, 2.5 to threefold higher expression was noted in syncBeWo cells in mono and co-culture (*P* < *0.05)* (Fig. 6D–F). In monoculture of all cell types we noted low protein expression of COMT. Expression of COMT was slightly increased at 72 h of co-culture in BeWo cells with H295R and was the highest in co-culture of syncBeWo cells with H295R (Fig. 6G–I).

## Discussion

In the presented data we compare different steroidogenic and metabolic parameters in co-cultures of JEG-3, BeWo and syncBeWo placental cell lines with an adrenal cell line (H295R).

By comparing co-cultures of placental cells we showed that both P4 and E2 secretion increased in JEG-3 and BeWo while in syncBeWo lower P4 secretion was noted. Lower levels of P4 secretion noted in co-culture of syncBeWo cells could be explained by the high levels of E2 secreted by the syncBeWo cells. It is well known that P4 levels during placentation decrease in favor of increased E2 secretion (Hill 2014) and diminished hCG production (Aspillaga et al. 1983), which we also observed in our study. Although the protein expression profile was similar in all tested models, the highest expression of 3βHSD was observed in co-culture of syncBeWo cells. It is well known that 3βHSD enzymes operate during placental cell differentiation (Meeker et al. 1971).

E2 secretion was significantly higher in all placental co-culture models compare to monocultures. The higher levels of E2 secretion can be explained with increased CYP19 protein expression in all co-culture models at all time points. Observed by us increased synergistically E2 secretion in co-culture of H295R and BeWo after 72 h of culture cells is in accordance with previously published data (Thibeault et al. 2014). It is also in agreement with data by Hill (2014), who showed that progression of development and differentiation of the placenta is connected with an increase of placental capacity for the aromatization of androgens, and therefore with an increase of CYP19 expression or aromatase activity.

Differences in hormone secretion observed in co-culture models were results from the interaction between placental and adrenal. For estrogen synthesis, in contrast to other steroidogenic organ, the placenta does not express the cytochrome P450 17β-hydroxylase-17:20 lyase, and therefore cannot convert pregnenolone and progesterone into androgens. Thus the production of placental estrogens is tributary of a precursor androgen, the sulphate of dehydroepiandrosterone (S-DHA) produced by the maternal and adrenal gland (Thibeault et al. 2014).

We have demonstrated the cell-specific secretion of hCG in both mono- and co-cultures. The highest level of secretion of this hormone was noted in the JEG-3 cell; however, there was no difference between secretion by mono- and co-culture. It has previously been shown that a large amount of hCG, directly involved in the quality of placentation, is secreted by extravillous trophoblasts during the first trimester of pregnancy and likely hyperglycosylated hCG secretion promotes trophoblast invasion (Handschuh et al. 2007), thus confirming our results. Additionally, we showed that syncBeWo cells secreted four times more hCG than BeWo cells which corresponded with lower P4 levels. As we discussed above, during placentation P4 levels decrease in favor of an increase in E2 secretion (Hill 2014) and diminished hCG production (Aspillaga et al. 1983).

No differences between PR expression in JEG-3 and BeWo co-culture, while statistically significant lower PR expression in co-cultures of syncBeWo cells were observed. The profile of PR protein expression, in co-culture of JEG-3 and BeWo, increased over time of cell culture, while there was no change in syncBeWo cells. Lower PR expression noted in co-culture of syncBeWo cell could be explained by lower P4 secretion corresponding with high levels of E2 secretion and ER expression. Zachariades et al. (2011) suggested that PR is implicated in the developmental events leading to placental syncytialisation. Our observation of the downregulation of PR expression in syncBeWo cell versus BeWo cells could be justified by the fact that these cells are fully syncytialised, thus resembling a third trimester placental model (Zachariades et al. 2011).

From our observation of ER, all used placental cell lines had higher expression of ERα than ERβ. We noted no significant difference between monoculture and co-culture of JEG-3 and BeWo cell models although there was higher ERα expression in syncBeWo. This finding is in agreement with the data of Bukovsky et al. (2003) who showed similar relationships in placental explants. Bukovsky et al. (2003) documented that E2 plays a role in the stimulation of terminal differentiation of mononucleated cytotrophoblast cells and promotes placental function, via the ERα. An increase of ERα/βγ mRNA in the placenta throughout gestation was reported by Fujimoto et al. (2005).

The next important problem discussed in this study relates to receptor and enzymes involved in the metabolism of different exogenous and endogenous compounds. The AhR acts as a xenobiotic receptor for a number of different xenobiotics, thus playing a critical role in human placenta (Stejskalova and Pavek 2011). We observed statistically significant differences in AhR expression between the used models with the highest expression in co-culture of syncBeWo. Taking into account that there are studies which show multiple mechanisms of AhR-ER (Helle et al. 2016; Barć and Gregoraszczuk 2016) we suggest that this difference between the types of model is attributed to higher ER expression in syncBeWo. The consequence of AhR receptor stimulation is the activation of detoxifying enzymes of phase I (CYP1A1) and phase II (i.e. COMT) of metabolism. In the present study, we showed comparable CYP1A1 and COMT expression in JEG-3 and BeWo cells, while higher expression was observed in syncBeWo cells, both in mono- and co-culture. CYP1A1 expression and activity have been detected in the first and third trimesters at both mRNA and protein levels (Hakkola et al. 1996a, b; Stejskalova and Pavek 2011; Czekaj et al. 2005). Palmer et al. (2011) analyzed COMT expression showed that it was mainly expressed in the syncytiotrophoblast, which confirms our result of a higher expression of COMT in syncBeWo. Higher expression of both CYP1A1 and COMT in syncBeWo cells could be additionally explained by the fact that both enzymes are important for the metabolism of estrogens (Parl et al. 2009; Mannisto and Kaakkola 1999), which were in our co-culture model produced at the highest amount in syncBeWo cells compared with JEG-3 and BeWo cells. The exact molecular mechanism underlying the difference between unsyncytialised and syncytialised cells needs further study however it was not a scope of our study.

## Conclusion

Based on our results we conclude that, **t**he difference in hCG secretion between JEG-3 and BeWo representing villous and extravillous phenotype could be used to study fetoplacental steroidogenesis in 1st and 3rd trimester, respectively. In turn syncBeWo model exhibiting the highest expression of AhR, CYP1A1 and COMT could be suitable to study the metabolism. However, taking into consideration that in the development and function of the human placenta participate numerous factors we must keep in mind the limitation of each experimental model.

## Electronic supplementary material

Below is the link to the electronic supplementary material.

Supplementary material 1 (DOCX 14 kb)

Supplementary material 2 (DOCX 16 kb)
