# Supplementary material for: Co-culture of JEG-3, BeWo and syncBeWo cell lines with adrenal H295R cell line: an alternative model for examining endocrine and metabolic properties of the fetoplacental unit
Source: Cytotechnology. 2017 Sep 30;70(1):285–97. doi: 10.1007/s10616-017-0142-z (PMC5809658; doi:10.1007/s10616-017-0142-z)
Supplement: Supplementary file 1 — Supplementary material 1 (DOCX 14 kb) [file 10616_2017_142_MOESM1_ESM.docx]

CYTOTECHNOLOGY

“Co-culture of JEG-3, BeWo and syncBeWo cell lines with adrenal H295R cell line: an alternative model for examining endocrine and metabolic properties of the fetoplacental unit.”

Eliza Drwal^1^, Agnieszka Rak^1^, Ewa Gregoraszczuk^1,2^

^1^Department of Physiology and Toxicology of Reproduction, Institute of Zoology and Biomedical Research, Jagiellonian University in Krakow, Krakow, Poland

^2^Correspondence to: ewa.gregoraszczuk@uj.edu.pl

**Tab.1**. Syncytin-1 *vs* GAPDH mRNA expression (RQ ± SD). Statistical difference between BeWo vs BeWo + forskolin was indicated *** *(P<0.001)*, while before and after trypsynization by ^#^ *(P<0.05).*

| Time | **Before trypsynization** | |
| --- | --- | --- |
|  | BeWo | BeWo + forskolin |
| 24 h | 1,070 ± 0, 041 | 23,709 ± 1,749^***^ |
| 72 h | 0,584 ± 0,081 | 30,713 ± 2,938^***^ |
| **After trypsynization** | | |
| 24 h | 1,012 ± 0,082 | 37, 346 ± 0,550^***#^ |
