# Supplementary material for: Co-culture of JEG-3, BeWo and syncBeWo cell lines with adrenal H295R cell line: an alternative model for examining endocrine and metabolic properties of the fetoplacental unit
Source: Cytotechnology. 2017 Sep 30;70(1):285–97. doi: 10.1007/s10616-017-0142-z (PMC5809658; doi:10.1007/s10616-017-0142-z)
Supplement: Supplementary file 2 — Supplementary material 2 (DOCX 16 kb) [file 10616_2017_142_MOESM2_ESM.docx]

CYTOTECHNOLOGY

“Co-culture of JEG-3, BeWo and syncBeWo cell lines with adrenal H295R cell line: an alternative model for examining endocrine and metabolic properties of the fetoplacental unit.”

Eliza Drwal^1^, Agnieszka Rak^1^, Ewa Gregoraszczuk^1,2^

^1^Department of Physiology and Toxicology of Reproduction, Institute of Zoology and Biomedical Research, Jagiellonian University in Krakow, Krakow, Poland

^2^Correspondence to: ewa.gregoraszczuk@uj.edu.pl

**Fig.1.** Alamar blue assay in monoculture of syncBeWo and co-culture of syncBeWo with H295R cells after trypsynization at 24 and 72 h of culture.
